# Complete genome sequence of a multidrug-resistant *Proteus mirabilis* clinical isolate harboring 22 antimicrobial resistance genes including *bla*_CTX-M-15_

**DOI:** 10.1128/mra.00592-26

**Published:** 2026-09-17

**Authors:** Tracy H. Hazen, Gwen L. Robinson, Indira French, Gwen Paszkiewicz, Sierra Spindler, Michael S. Humphrys, Surbhi Leekha, J. Kristie Johnson, L. Leigh Smith

**Affiliations:** 1 Center for Advanced Microbiome Research and Innovation, University of Maryland School of Medicine 12264 , Baltimore, Maryland, USA; 2 Institute for Genome Sciences, University of Maryland School of Medicine 12264, Baltimore, Maryland, USA; 3 Department of Microbiology and Immunology, University of Maryland School of Medicine 12264 , Baltimore, Maryland, USA; 4 Department of Epidemiology and Public Health, University of Maryland School of Medicine 12264 , Baltimore, Maryland, USA; 5 Department of Pathology, University of Maryland School of Medicine 12264 , Baltimore, Maryland, USA; Fluxus Inc., Sunnyvale, California, USA

**Keywords:** *Proteus mirabilis*, antimicrobial resistance, wound infection, clinical

## Abstract

*Proteus mirabilis* causes urinary tract infections and wound infections and frequently exhibits multidrug resistance, complicating patient treatment. Here, we describe the complete genome sequence of a multidrug-resistant *P. mirabilis* wound isolate from 2025, providing insight into the repertoire of antimicrobial resistance genes in a recent *P. mirabilis* clinical isolate.

## ANNOUNCEMENT

*Proteus mirabilis* is a gram-negative bacterium that can be found in soil, water, and wastewater, as well as colonizing the gastrointestinal tract of animals and humans (1). *P. mirabilis* is also a versatile opportunistic pathogen that causes human urinary tract infections, wound infections, and bacteremia (2, 3). Prior studies reported an increase of drug-resistant *P. mirabilis* among hospitalized patients (4, 5).

*P. mirabilis* isolate Pm879 was obtained in April 2025 from a right leg soft tissue wound of a hospitalized patient who presented with gangrene. A deep wound sample was collected using a swab and cultured on MacConkey agar and blood agar. The isolate was stored at −80°C. Species identification was performed using VitekMS (Biomérieux). Antimicrobial susceptibilities were determined using MicroScan WalkAway panel NM56 (Beckman Coulter) and interpreted with Clinical and Laboratory Standards Institute breakpoints (6). Multidrug resistance was determined as resistance to three or more antimicrobial categories (7). Pm879 was grown on blood agar overnight at 37°C, and genomic DNA was extracted using the Nanobind CBB kit (Pacific Biosciences). The quantity and sizing of the genomic DNA were determined by fluorometric detection and Femto Pulse electrophoresis (Agilent). Sequencing libraries were generated without size selection using the Rapid Barcoding Kit (SQK-RBK114.24) (Oxford Nanopore Technologies). The genome of Pm879 was sequenced on a MinION MK1D (Oxford Nanopore Technologies) using R10.4.1 flow cells, generating 324,737 total reads with an N50 of 20,985 bp, yielding 585× sequence coverage. Basecalling, adapter trimming, and index trimming were performed using MinKNOW v.25.05.14 with the Dorado basecall server v.7.9.8 (https://nanoporetech.com/software/other/dorado-basecall-server) and the dna_r10.4.1_e8.2_400bps_sup@v5.2.0 model. Reads that passed QC (Q > 10) were assembled using Autocycler v.0.5.2 (8) with default settings. This generated a resolved consensus assembly with a single circular chromosome and two circular plasmids, which had 100% completeness and <1% contamination determined using CheckM2 v.1.1.0 (9). Sequences were not reoriented. Taxonomic classification was performed using GTDB-Tk v.2.5.2 (10, 11). Annotation by PGAP v.6.10 (12) predicted 4,129 total genes, including 3,932 protein-coding sequences and 108 RNA genes.

The genome of Pm879 contains a single chromosome with a sequence length of 4,340,484 bp and G + C content of 39.5%. The average nucleotide identity of Pm879 compared to the RefSeq reference genome of *P. mirabilis* (GCF_000069965.1) was 98.74%, which is greater than the ≥95% threshold for species-level classification (13). Pm879 also contains two plasmids with sequence lengths of 35,988 bp (GC 34.3%) and 25,868 bp (GC 40.2%), identified using MOB-suite v.3.1.9 (14). Multilocus sequence typing using PubMLST (15) identified Pm879 as sequence type (ST) 446. Previous ST446 clinical isolates have been described with acquired carbapenem resistance (16) and aztreonam-avibactam resistance (17). AMRFinderPlus v.4.2.7 (18) identified 22 resistance genes in the chromosome of Pm879 (Table 1). A prior study demonstrated *P. mirabilis* isolates in a widespread global lineage contained, on average, 17 antimicrobial resistance genes, while other lineages had ~8 to 12 resistance genes per isolate (19). The large number of antimicrobial resistance genes of Pm879 (>22) highlights the importance of genomic surveillance to monitor acquired drug resistance mechanisms among pathogens infecting and colonizing hospitalized patients.

**TABLE 1:** Antimicrobial susceptibilities and resistance determinants of Pm879^*a*^

Antibiotic	Drug class	Resistance genes	MIC (µg/mL)	Interpretation
Amikacin	Aminoglycosides	*aadA1, aph(3′)-Ia, aph(3″)-Ib, aph(6)-Id, aac(3)-IIe, ant(2″)-Ia, aac(6′)-Ib, aac(6′)-Ib-cr5*	≤16	S
Gentamicin			>8	R
Tobramycin			>8	R
Ampicillin	Beta-lactams	*bla*_TEM-1_, *bla*_OXA-1_, *bla*_CTX-M-15_	>16	R
Aztreonam			>16	R
Ampicillin-sulbactam	Beta-lactams + beta-lactamase inhibitors	*bla*_CTX-M-15_, *bla*_OXA-1_	16/8	I
Pipercillin-tazobactam			≤8	S
Cefazolin	Cephalosporins	*bla*_CTX-M-15_	>16	R
Cefepime			>16	R
Ceftriaxone			>32	R
Ertapenem	Carbapenems	None	≤0.5	S
Meropenem			≤1	S
Trimethoprim-sulfamethoxazole	Dihydrofolate reductase inhibitor- sulfonamide	*dfrA1, sul1, sul2*	>2/38	R
Levofloxacin	Fluoroquinolones	*qnrB1, aac(6′)-Ib-cr5*	4	R
Ciprofloxacin			>2	R
Tetracycline	Tetracyclines	*tet(A), tet(J*)	>8	R

^*a*^ Additional resistance determinants harbored by Pm879 for which susceptibilities were not determined: *sat2, catA1, catB3, catA*, and *qacEdelta1*.

## Data Availability

The sequence data from *P. mirabilis* isolate 879 are deposited under BioProject PRJNA1445273 and BioSample SAMN56782725. The raw sequence reads are deposited in SRA under SRS28915757 and the genome assembly is deposited in GenBank under JBWQEX000000000.

## References

[1] Armbruster CE, Mobley HLT, Pearson MM. 2018. Pathogenesis of *Proteus mirabilis* infection. EcoSal Plus 8. doi:10.1128/ecosalplus.ESP-0009-2017 PMC5880328 29424333

[2] Armbruster CE, Mobley HLT. 2012. Merging mythology and morphology: the multifaceted lifestyle of *Proteus mirabilis*. Nat Rev Microbiol 10:743–754. doi:10.1038/nrmicro2890 PMC3621030 23042564

[3] Chakkour M, Hammoud Z, Farhat S, El Roz A, Ezzeddine Z, Ghssein G. 2024. Overview of *Proteus mirabilis* pathogenicity and virulence. Insights into the role of metals. Front Microbiol 15:1383618. doi:10.3389/fmicb.2024.1383618 PMC11026637 38646633

[4] de Almeida LKS, Silva LC, Guidone GHM, de Oliva BHD, do Nascimento AB, Faustino G, da Silva Pimenta J, Vespero EC, Rocha SPD. 2025. Impact of COVID-19 pandemic on antimicrobial resistance of *Proteus mirabilis* in a Brazilian hospital. Braz J Microbiol 56:499–510. doi:10.1007/s42770-024-01568-8 PMC11885744 39630218

[5] Facciolà A, Gioffrè ME, Chiera D, Ferlazzo M, Virgà A, Laganà P. 2022. Evaluation of antibiotic resistance in *Proteus* spp: a growing trend that worries public health. results of 10 years of analysis. New Microbiol 45:269–277. 36190369

[6] CLSI. 2023. CLSI supplement M100. *In* Performance standards for antimicrobial susceptiblity testing, 33rd ed. The Clinical and Laboratory Standards Institute (CLSI).

[7] Magiorakos AP, Srinivasan A, Carey RB, Carmeli Y, Falagas ME, Giske CG, Harbarth S, Hindler JF, Kahlmeter G, Olsson-Liljequist B, Paterson DL, Rice LB, Stelling J, Struelens MJ, Vatopoulos A, Weber JT, Monnet DL. 2012. Multidrug-resistant, extensively drug-resistant and pandrug-resistant bacteria: an international expert proposal for interim standard definitions for acquired resistance. Clin Microbiol Infect 18:268–281. doi:10.1111/j.1469-0691.2011.03570.x 21793988

[8] Wick RR, Howden BP, Stinear TP. 2025. Autocycler: long-read consensus assembly for bacterial genomes. Bioinformatics 41:btaf474. doi:10.1093/bioinformatics/btaf474 PMC12460055 40875535

[9] Chklovski A, Parks DH, Woodcroft BJ, Tyson GW. 2023. CheckM2: a rapid, scalable and accurate tool for assessing microbial genome quality using machine learning. Nat Methods 20:1203–1212. doi:10.1038/s41592-023-01940-w 37500759

[10] Chaumeil PA, Mussig AJ, Hugenholtz P, Parks DH. 2022. GTDB-Tk v2: memory friendly classification with the genome taxonomy database. Bioinformatics 38:5315–5316. doi:10.1093/bioinformatics/btac672 PMC9710552 36218463

[11] Parks DH, Chaumeil PA, Mussig AJ, Rinke C, Chuvochina M, Hugenholtz P. 2026. GTDB release 10: a complete and systematic taxonomy for 715 230 bacterial and 17 245 archaeal genomes. Nucleic Acids Res 54:D743–D754. doi:10.1093/nar/gkaf1040 PMC12807784 41123020

[12] Tatusova T, DiCuccio M, Badretdin A, Chetvernin V, Nawrocki EP, Zaslavsky L, Lomsadze A, Pruitt KD, Borodovsky M, Ostell J. 2016. NCBI prokaryotic genome annotation pipeline. Nucleic Acids Res 44:6614–6624. doi:10.1093/nar/gkw569 PMC5001611 27342282

[13] Jain C, Rodriguez-R LM, Phillippy AM, Konstantinidis KT, Aluru S. 2018. High throughput ANI analysis of 90K prokaryotic genomes reveals clear species boundaries. Nat Commun 9:5114. doi:10.1038/s41467-018-07641-9 PMC6269478 30504855

[14] Robertson J, Nash JHE. 2018. MOB-suite: software tools for clustering, reconstruction and typing of plasmids from draft assemblies. Microb Genom 4:e000206. doi:10.1099/mgen.0.000206 PMC6159552 30052170

[15] Jolley KA, Bray JE, Maiden MCJ. 2018. Open-access bacterial population genomics: BIGSdb software, the PubMLST.org website and their applications. Wellcome Open Res 3:124. doi:10.12688/wellcomeopenres.14826.1 PMC6192448 30345391

[16] Sattler J, Noster J, Stelzer Y, Spille M, Schäfer S, Xanthopoulou K, Sommer J, Jantsch J, Peter S, Göttig S, Gatermann SG, Hamprecht A. 2024. OXA-48-like carbapenemases in *Proteus mirabilis* – novel genetic environments and a challenge for detection. Emerg Microbes Infect 13:2353310. doi:10.1080/22221751.2024.2353310 PMC11123474 38712879

[17] Guo J, Wu C, Tang X, Wang L, Qi Y, Yu Y, Tu Y, Zhang L, Li X. 2026. Genomic epidemiology and aztreonam–avibactam resistance mechanisms of *Proteus mirabilis* in China: an eight-year retrospective study. Ann Clin Microbiol Antimicrob 25:15. doi:10.1186/s12941-026-00852-9 PMC12955263 41630025

[18] Feldgarden M, Brover V, Gonzalez-Escalona N, Frye JG, Haendiges J, Haft DH, Hoffmann M, Pettengill JB, Prasad AB, Tillman GE, Tyson GH, Klimke W. 2021. AMRFinderPlus and the reference gene catalog facilitate examination of the genomic links among antimicrobial resistance, stress response, and virulence. Sci Rep 11:12728. doi:10.1038/s41598-021-91456-0 PMC8208984 34135355

[19] Zhang T, Wei H, Ju Z, Wang Q, Li J, Wen R, Wu J, Wang H, Lei C. 2026. Genomic survey uncovers the emergence of a multidrug-resistant dominant lineage in *Proteus mirabilis* populations. NPJ Antimicrob Resist 4:17. doi:10.1038/s44259-026-00189-5 PMC12957351 41776319

